# Two- and Three-dimensional Rings in Drugs

**DOI:** 10.1111/cbdd.12260

**Published:** 2014-01-01

**Authors:** Matteo Aldeghi, Shipra Malhotra, David L Selwood, Ah Wing Edith Chan

**Affiliations:** 1 Wolfson Institute for Biomedical Research, University College LondonGower Street, London, WC1E 6BT, UK

**Keywords:** drug, fragment, lead optimization, ring

## Abstract

Using small, flat aromatic rings as components of fragments or molecules is a common practice in fragment-based drug discovery and lead optimization. With an increasing focus on the exploration of novel biological and chemical space, and their improved synthetic accessibility, 3D fragments are attracting increasing interest. This study presents a detailed analysis of 3D and 2D ring fragments in marketed drugs. Several measures of properties were used, such as the type of ring assemblies and molecular shapes. The study also took into account the relationship between protein classes targeted by each ring fragment, providing target-specific information. The analysis shows the high structural and shape diversity of 3D ring systems and their importance in bioactive compounds. Major differences in 2D and 3D fragments are apparent in ligands that bind to the major drug targets such as GPCRs, ion channels, and enzymes.

The success 1 in the last decade of fragment-based drug discovery (FBDD) has led to the screening and design of small fragments being common practice in many drug discovery programs 2. The selection of fragment libraries is usually done on the basis of molecular physical and chemical properties, such as the rule of three 3,4. With the increasing employment of diversity-oriented synthesis (DOS), attention has focused on fragments' skeletal diversity and in particular on their degree of saturation and structural, or shape, complexity 5,6. Saturation and complexity are the two main properties that define the so-called ‘3D fragments’ 7,8. Fragments comprising a saturated core, with sp^3^ carbon atoms, are generally considered to have a three-dimensional character, as opposed to planar or flat aromatic scaffolds. The saturated core offers the possibility to access more globular shapes and vectors for fragment expansion.

Aromatic ring scaffolds are often used in medicinal chemistry as they have more robust chemistry than 3D scaffolds. A variety of aromatic reagents are readily available and they tend to be tractable substrates that can be systematically modified. Aromatic synthetic paths are well characterized, with a number of metal-mediated couplings in order to create aryl-aryl systems and C-C bond forming reactions. For example, Suzuki and Sonogashira couplings, Friedel-Crafts acylation and alkylation, are reactions routinely used to selectively modify aromatic systems. Saturated rings, however, are harder to substitute at the desired positions and tend to rely on heteroatom alkylation and arylation. C-C bond-forming reactions are less common and often generate stereocenters. Roughley and Jordan have indeed noticed the presence of at least one aromatic ring in 99% of the compounds present in their dataset of medicinal chemistry reactions 9. Fragment-based drug discovery (FBDD) has often focused on sp^2^-rich aromatic compounds. However, molecules with aromatic scaffolds can impair shape diversity, and possibly limit the exploration of a subset of biological space. Sauer *et al*. suggest that a screening library designed to contain molecules with high molecular shape diversity would be expected to show a broader range of biological activities 10. Hung *et al*. proposed that DOS could be used to generate fragments with enriched sp^3^ carbon content and fragments with increased 3D character would give access to a larger chemical space as compared to those libraries currently in use, and would thus possibly allow addressing ‘difficult’ targets 5.

Lovering *et al*. 7 analyzed the effect of saturation and complexity on molecule solubility and compound progression through the drug development stages. The researchers found that a higher proportion of sp^3^-hybridized carbons was associated with increased solubility and with compound success from discovery, through clinical testing, to drugs. Similarly, researchers from GSK 11 suggested that aromatic ring count negatively affects the developability properties of lead compounds, being correlated with lower solubility and higher lipophilicity, along with other undesirable properties. Taken together, these features can potentially lead to poorer efficacy and greater toxicity. Conversely, hetero-aliphatic ring count correlated with more desirable properties, such as increased solubility, decreased lipophilicity, and decreased albumin-binding and cytochrome P450 inhibition 11,12. Clemons *et al*. screened three sets of compounds, characterized by different degrees of saturation and structural complexity, against 100 sequence-unrelated proteins using small-molecule microarrays. They found that increasing the content of sp^3^-hybridized atoms improved binding selectivity and frequency, therefore resulting in improved performance of screening collections 13. In a later follow-up analysis, Clemons and colleagues, although clearly stating that a causative relationship is not supported by their results, suggested that specific binders tend in fact to have more 3D character than promiscuous compounds 14.

The success of FBDD is considerably dependent on the quality of the fragment library used for screening; not only the properties of the fragments but also what protein targets they are screened for. As suggested by the studies mentioned above, employing more 3D fragments might increase the diversity of fragment screening libraries, improve their ADMET properties, and lead to better starting points for lead generation and optimization. Rings and ring systems play an essential role in drug scaffolds 15. They represent systems of atoms where many degrees of freedom are removed, give molecules their fundamental shape, determine in large part the degree of flexibility of the compound and the position of the side chains. A detailed analysis of the 2D and 3D fragments found in approved drugs, together with their intended protein targets can give further insights to the role of these fragments in drug binding.

In this study, we define a fragment to be a contiguous system of rings, i.e., all rings sharing at least one atom. In addition, a 3D fragment must contain at least one sp^3^ hybridized carbon atom. Conversely, fragments with no sp^3^ hybridized carbon atoms are defined as 2D. A simple classification like this is easy to remember and would enable scientists to use the information and put it into practice. In addition, this system easily separates ring systems that are totally flat (2D). Figure 1 illustrates the definition of 2D and 3D fragments with the protease inhibitor Indinavir. A further subset, 3D-h within 3D fragments is defined as 3D hybrid/fused ring system containing both pure 3D and aromatic rings. Comparison of 2D and 3D rings was carried out with the use of shape and saturation descriptors plus other property indices. The analysis also takes into account the classes of their protein target, providing additional information and recording potential target-dependent preferences toward flat or three-dimensional rings. This data show the importance of 3D ring systems in bioactive compounds, and indicates significant 2D/3D ring preferences among different protein families.

**Figure 1:**
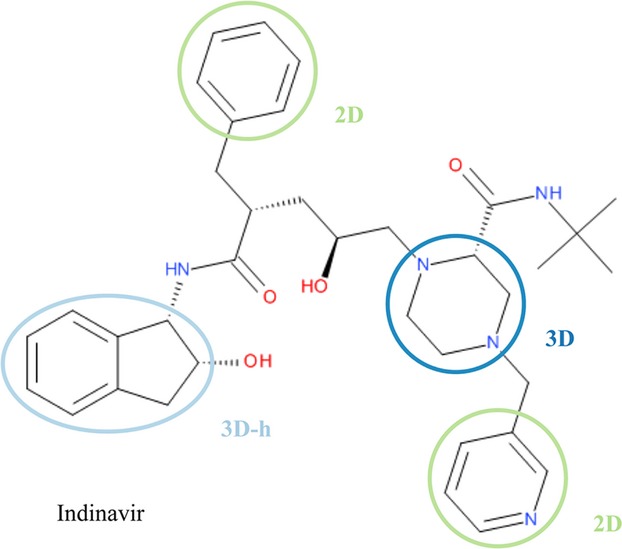
Definition of 2D and 3D ring fragments, using Indinavir as an example. A ring fragment is defined as a ring assembly. Fragments with no sp^3^ carbon are classed as 2D (green), the rest as 3D (blue). 3D-h (light blue) is a subset of the 3D fragments, denoting 3D hybrid/fused ring systems containing both pure 3D and aromatic rings.

## Methods and Materials

### Drug data set

The set of FDA approved drugs (version 19 March 2012) was retrieved from DrugBank 16. The following structures were filtered out using Pipeline Pilot 8.5: drugs which are nutraceuticals or contain elements other than carbon, nitrogen, oxygen, phosphorus, sulfur, fluorine, chlorine, bromine, or iodine. The usual chemical and physical property filters, such as molecular weight or hydrogen bond count, were not employed on this set of data. A final set of 1297 drugs was obtained and they have the following properties: molecular weight ranges 30.0–1736.2; AlogP -18.7–14.2; hydrogen bond donors 0–37; hydrogen bond acceptors 0–44; number of rotatable bonds 0–44.

### Ring fragment definition

Pipeline Pilot 8.5 was used to generate ring fragments from the molecules. The definition of ring assemblies, including exocyclic double bonds, was used in the component ‘Generate Fragments’. It generates contiguous ring systems that share one or more atoms, including alpha atoms only if they double bond with an atom that is part of the ring (e.g., carbonyls in lactam compounds). Fragments with no sp^3^ carbon atoms were defined as ‘2D fragments’ while fragments with at least one sp^3^ carbon atom were defined as ‘3D fragments’ as illustrated in Figure 1. Three dimensional-h (3D-h) is a subset of 3D fragments denoting 3D hybrid/fused ring system containing both pure 3D and aromatic rings. The number of sp^3^ hybridized carbons and total carbon count were calculated using Pipeline Pilot 8.5.

### Molecular similarity

An all-by-all Tanimoto matrix, built on Pipeline Pilot's functional-class fingerprints (FCFP_4), was generated for the non-redundant set of fragments to assess their molecular similarity. Such a matrix is difficult to interpret and visualize. Hence, a multidimensional scaling (MDS) technique 17 was employed to extract the key information from this matrix. MDS is a method whereby distance data can be visualized in a small number of dimensions, e.g., a 2D or 3D map. The points representing molecular fingerprints are arranged in the 2D plane in such a way that the root-mean-square change in the distance when going from the original matrix to the new representation is minimized. A computer program employing this technique was written in-house in the C programming language. To compare the similarity of fragments within different protein targets, the pairwise Tanimoto index between each fragment was separated for each target class, generating 15 matrices for the 15 target classes studied here. A Kruskal–Wallis one-way analysis of variance (K–W test) was performed using these fifteen matrices, using the all-by-all matrix for all non-redundant fragments as reference. This K–W test is a non-parametric version of the one-way analysis of variance and an extension of the Wilcoxon rank sum test for more than two groups, and it compares the medians of the data given as input in order to determine whether the samples come from the same population. A significant result in the K–W test means at least one sample comes from a different population; however, it does not allow pair comparison between samples. In order to estimate which groups (i.e., target classes) are significantly different from others, a multiple comparison test is needed. Therefore, Tukey's honestly significant difference method was used as *post hoc* analysis in order to compare the samples. All statistics were performed using Matlab R2012b.

### Molecular shape descriptor

Principal moments of inertia (PMI) ratios were used as described by Sauer *et al*. as an indication of the molecular shape of the fragments 10. The three PMIs (I_1_, I_2_, I_3_; where I_3_ is the largest diagonal value) were calculated with Pipeline Pilot 8.5, and a two-dimensional map was built plotting the ratios I_1_/I_3_ versus I_2_/I_3_. A two-sample Kolmogorov–Smirnov test (in Matlab) was also used in order to compare the samples.

### Target class assignment

Data on the biological targets of marketed drugs were manually curated, starting from information retrieved from DrugBank, EBI DrugPort, and Overington *et al*. 18. Fifteen classes were defined. Six enzyme classes according to the reaction they catalyze: oxidoreductases, transferases, hydrolases, lyases, isomerases, and ligases; and three receptor classes: G-protein coupled receptors (GPCR), nuclear receptors, and other receptors. The rest were ion channels, penicillin-binding proteins, transporters, cellular proteins, nucleic acids and ‘others’. Only the top level of family classification was used in the study as the statistics are only meaningful at the superfamily level. Each of the 1191 drugs (of the 1232 drugs containing rings) has one target class assignment. The 41 drugs remaining are either chelating or contrast agents or simply did not report any protein target in DrugBank.

## Results and Discussion

### Ring fragment profile

**Figure 2:**
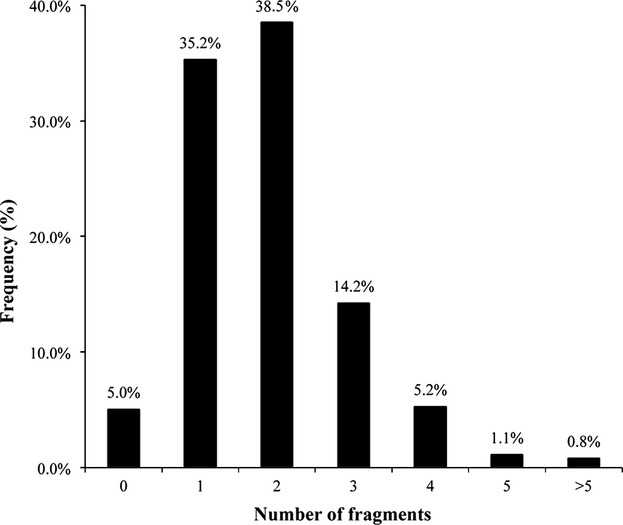
Ring fragment count distribution in our dataset of marketed drugs.

Only 65 of 1297 marketed drug molecules (about 5%) have no ring structure, showing how important ring assemblies are in drug skeletons. After ring fragmentation, the remaining 1232 drug molecules have a total of 433 unique and non-redundant ring fragments, consisting of 101 2D and 332 3D fragments. Within the 3D fragments, 135 are 3D hybrid ring systems (3D-h). Figure 2 plots the distribution of the number of ring fragments in drug molecules. The majority of drugs (74%) contain one or two ring assemblies. Fewer than 2% of the drug molecules have five rings or more. This is not surprising as the molecular weight (thus loosely related to number of ring fragments in the drugs) of most drugs tend not to exceed 500 as observed by Lipinski's Ro5 19.

**Figure 3:**
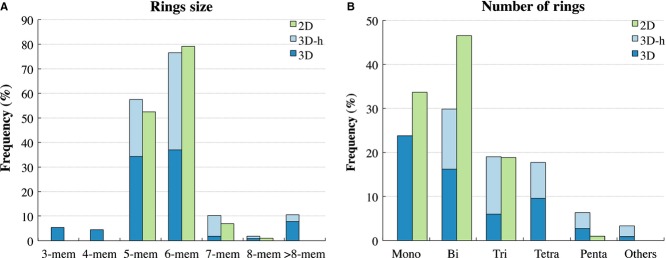
Ring type distributions among 2D and 3D ring fragments. (A) percentage of ring fragments containing at least one ring of the specified size; e.g., 76.5% of 3D fragments and 79.2% of 2D fragments contain at least one 6-membered ring. (B) percentage of ring fragments that are composed by one, two, three, four, five, or more rings.

The list of 2D and 3D ring fragments is provided in supplementary Table S1. The ratio of 2D to 3D rings is about 1 to 3. There are in fact more unique 3D ring structures than 2D in this drug dataset. This could be because a saturated ring can allow a larger variety of structures; e.g., in our data set there are saturated 3-membered rings, such as cyclopropane or oxirane, whereas unsaturated rings of this size are usually poorly stable. Among all fragments, bicyclic and spiro-cyclic ring motifs are the most frequent (146 cases), followed by 113 cases of monocyclic ring systems and 82 cases of tricyclic ring systems. Figure 3 plots the distributions of the nature of the rings as well as ring size. In 3D fragments mono-, bi-, and tri-cyclic rings represent 73% of fragments; there is in fact a higher presence of more complex systems, made by four, five or more rings, accounting for more than a quarter of all assemblies. Three dimensional fragments provide more variety. Of the 332 three-dimensional assemblies, 197 (∽60%) are pure 3D ring system, i.e., no fused aromatic ring at all. As shown in Figure 3, systems containing only 3D single rings and hybrid systems have relatively similar distributions of number of rings within their assemblies and ring sizes. Hybrid systems by definition cannot be monocycles and tend to have slightly larger assemblies. 5- and 6-membered are the most frequently occurring rings for both hybrid and 3D-only systems, both being found in more than half of the assemblies, though 6-membered rings absolutely dominate in hybrid systems, where, due to the high frequency of the benzene motif, 131 of 135 3D-h fragments contain at least one benzene motif. However, 3D-h systems do not contain 3- and 4-membered rings. The absence of small rings is expected considering unsaturated cycles of this size are very unstable and, conversely, aliphatic 3- and 4-membered rings are already strained systems that would be subjected to considerable restraints if fused with an aromatic cycle. Thus, the presence of these small rings among 3D fragments is entirely due to 3D-only systems.

The most popular 2D fragments are mono-, bi-, and tri-cyclic rings covering 99% of all cases. In flat ring systems, five- and six-membered rings are the most frequent, occurring respectively in about 52% and 79% of all 2D fragments. Also in three-dimensional fragments five and six-membered rings are the most frequent (occurring respectively in about 58% and 76% of fragments). However, there are also three-membered and four-membered cycles. Seven-membered rings are represented in about 10% of 3D fragments with a similar proportion of more-than-eight-membered rings. This incidence of large-sized rings is due to the presence of natural compounds such as cyclic peptides (e.g., Anidulafungin and Colistin). Considering aromaticity and sp^2^ hybridization impose considerable limitations on the number and arrangement of atoms in a ring, it is noteworthy that 2D and 3D ring systems differ more in the number of rings present in the assembly, which is not affected by the aromatic character, rather than mere ring size. This might suggest that systems made of four or five rings, where conjugation is broken by the presence of tetrahedral carbons, may be better tolerated than similar systems where many unsaturated rings are joined together.

**Figure 4:**
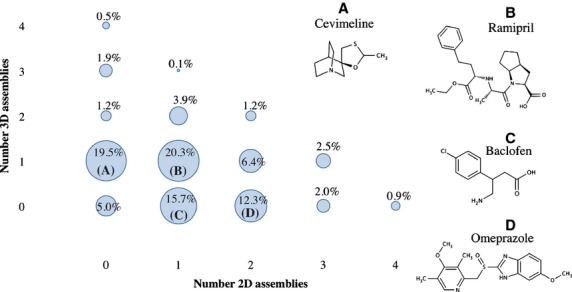
Combinations of 2D and 3D rings found in marketed drugs. The graph shows the percentages of the most common combinations of 2D and 3D assemblies. These associations represent 93.4% of the whole data set. On the right, A–D are examples of drug molecules taken from the respective 2D/3D fragment combination groups; e.g. B, Ramipril is a drug formed by one 2D and one 3D fragment.

To probe the 2D/3D combinations of the assemblies, Figure 4 displays the combinations of 2D and 3D ring systems found in marketed drugs. Four categories contribute most frequently: drugs that have only one 3D ring fragment (19.5%, e.g., Cevimeline); two ring fragments, one 2D and 3D ring fragment each (20.3%, e.g., Ramipril); one 2D ring fragment (15.7% e.g., Baclofen); and two 2D fragments (12.3%, e.g., Omeprazole). These four cases cover almost 70% of all the possible ring system combinations in drugs. Overall, 32.9% of all drugs contain only 2D ring assemblies, whereas 29.8% contain only 3D assemblies. Taken together, these results suggest that in successful bioactive compounds the proportion of flat ring systems to three-dimensional, geometrically more complex, aliphatic rings systems is in fact quite balanced, despite the general notion that medicinal chemists tend to over employ aromatic rings with the intention of improving potency.

**Figure 5:**
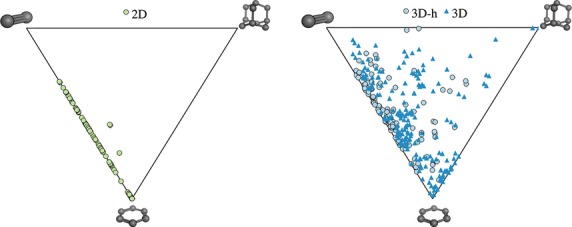
Principal moments of inertia (PMI) plot describing the molecular shape for the rings derived from marketed drugs. Two dimensional rings on the left and 3D rings on the right. Notice the more diverse distribution of shapes for 3D rings as compared to 2D.

In order to qualitatively evaluate the shape diversity of the 2D and 3D fragments, we used PMI descriptors. In fact, the sp^3^ carbon content does not directly describe the actual shape of molecules and does not address the question of whether our 2D and 3D rings in fact show a clear-cut difference in molecular shape. The result is a triangular map (Figure 5) where the three corners represent distinctive shapes, namely rod shapes (top-left corner) like acetylene, disk shapes (lowest corner) like benzene, and sphere shapes (top-right corner) like adamantane. Note that the line between the corners that stand for the rod and disk shapes represents flat compounds. The map only describes molecular shapes, without considering any other property; thus, benzene and pyridine essentially occupy the same position on the plot due to their very similar shape (an imperceptible difference in the position is due to the different mass of the nitrogen and carbon atoms, influencing their PMIs), albeit having different chemical properties. We assume that the further from the line a molecule is, the less flat and more ‘three-dimensional’ it is. As expected, 2D fragments are placed on the ‘flat line’, with the exception of two outliers due to the presence of sulfonyl groups. However, 3D fragments are scattered all over the map, suggesting a much higher assorted distribution of molecular shapes. In particular, the distribution of 3D-only assemblies seems to be more shifted toward a globular shape as compared to the 3D-h system (p = 7.2 × 10^−14^ for a two-sample Kolmogorov–Smirnov test between the two sets of geometric distances from the top-right corner.) The small p-value from the Kolmogorov–Smirnov test confirms that the presence of aromatic rings will impact on the globular shapes of molecules. As suggested by Sauer *et al*., although shape, together with size, is probably one of the most basic and first levels in the hierarchy of molecular descriptors, molecular shape diversity is a prerequisite for broad bioactivity of screening libraries 10. The map therefore suggests that 3D, aliphatic ring fragments have much to offer in terms of molecular shape diversity; 2D aromatic systems tend to offer less diversity, not only in terms of structure but also in shape.

### Fragments by target class

**Figure 6:**
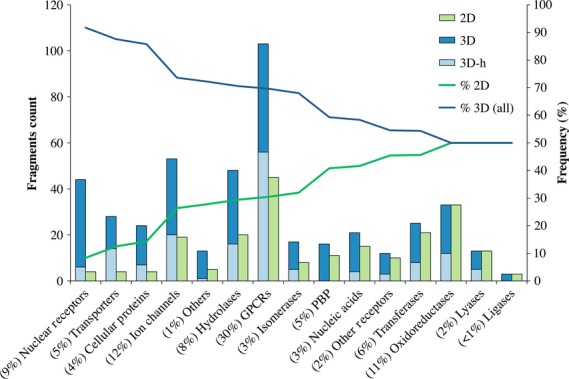
Number and percentage of 2D and 3D rings per each target class. Values in parentheses next to the target categories are the percentage distribution of targets among the marketed drugs analyzed. On the abscissa, the number of rings (left) and their percentage (right) in the target class.

Target information was initially retrieved from the DrugBank database and EBI DrugPort and then manually curated to a final 15 target categories, with a single class assigned to each drug as in Figure 6. The percentage distribution (the values next to the target classes in Figure 6) of targets among the marketed drugs set analyzed is similar to a previous study 18. Currently, the most common targets for small molecule drugs are GPCRs (356 drugs), followed by ion channels (138 drugs), and nuclear receptors (102 drugs).

With the above information, target classes were assigned to fragments, based on the drugs containing them. Figure 6 plots the number of fragments as well as the percentage of 2D and 3D fragments against their protein targets. Some target classes, such as GPCRs and ion channels, have more fragments associated with them compared to others (e.g., ligases and transporters). This is because, as shown by Figure 6, currently there are more GPCR or ion channels drugs than ligase or transporter ones. In Figure 6, target classes are ordered in descending 3D fragment percentage content, so it is possible to see the 3D fragment's contribution. For instance, among the fragment structures derived from nuclear receptors, 91.7% are 3D fragments, whereas among the structures obtained from drugs targeting oxidoreductases only 50% are 3D assemblies. In another example, if only the number of 3D fragments are counted, the highest numbers are 103 in GPCRs, followed by 53 in ion channels, 48 in hydrolases, and 44 in nuclear receptors. However, in terms of percentages, strikingly, the highest percentages of 3D fragments are found for 92% in nuclear receptors, 88% in transporters, and 86% in cellular proteins. Only four target classes, all enzymes (ligases, lyases, oxidoreductases, and transferases), have an even distribution of 2D and 3D fragments in their drugs. Nevertheless, no target class has a higher 2D contribution than 3D.

Within the 3D sets in Figure 6, the 3D-only systems represent the vast majority within a target class. For example, in nuclear receptors, penicillin-binding proteins, and nucleic acids, 3D-only systems represent more than 80% of 3D assemblies. The class of transporters shows instead a balance between the two 3D fragment subtypes, with exactly 50% of 3D-only and 50% of 3D-h. It seems that there is no correlation between the percentage of 2D fragments within a target class and the percentage of hybrid systems within the 3D fragments of that class. This data give further indication about what type of 3D ring systems might be favored by a specific target class. However, care should be taken as such target classes are very broad and often contain a number of diverse protein families. The implications of the size of the target classes will be later discussed along with an analysis of fragment similarity within each class, in order to highlight some possible caveats of the study and be able to critically assess the results. The ten most common fragments (i.e., the ten most frequently occurring structures) shown in Table 1, are associated with a high number of target classes. The fragment phenyl, **1**, exists in drugs that target all protein classes; with the only exception being ligases, although in our set of marketed drugs there are only 3 drugs targeting ligases, none of them having phenyl. On the other hand, fragment **10**, a well-known motif of *β*-lactam antibiotics, is associated with only one target class: penicillin-binding proteins. Another example of a ring system targeting only one class of protein, in spite of the fact it is found in many different compounds, is fragment **25** (Table 2), the core of benzodiazepines, which occurs in 14 different drug molecules but targets specifically ligand-gated ion channels (more precisely GABA_A_). Fragment **12** (a tricyclic), another common and well-known 2D fragment, was found 25 times in 25 different drugs. For example, in antipsychotic agents targeting dopamine receptors, such as Prochlorperazine and Propiomazine; antihistamine drugs, such as Aceprometazine and Trimeprazine; and the drug Moricizine targeting the sodium channel protein Na_v_1.5.

**Table 1:** The ten most frequent ring fragments found in marketed drugs

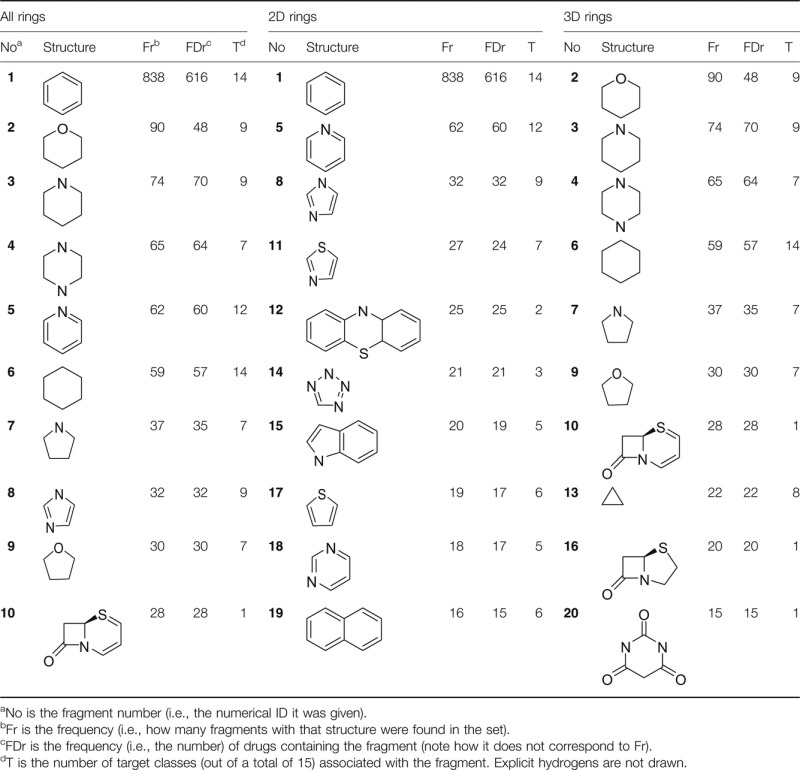

**Table 2:** Ring fragments occurring in at least five drugs while being associated to a single target class

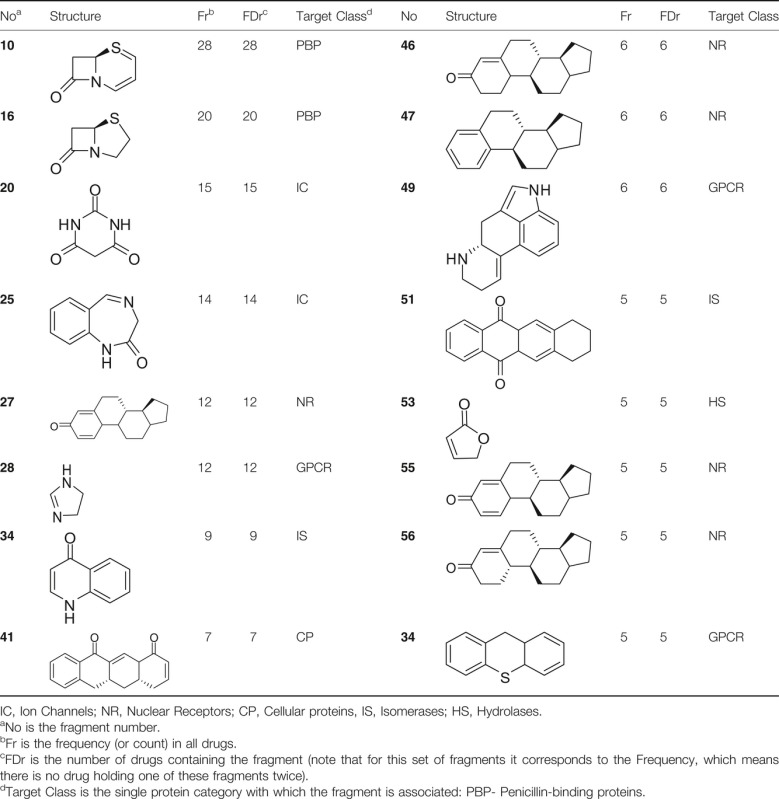

The results for the most frequent 3D fragments tell us that hetero-aliphatic rings are more frequently employed than the respective carbo-aliphatic rings with the same dimensions. This observation is also in accordance with Ritchie *et al*., who found higher hetero-aliphatic than carbo-aliphatic ring counts in GSK medicinal chemistry compounds 12. The factors contributing to such a discrepancy can be different, from ease of synthesis to, more importantly, physicochemical properties. Aliphatic rings containing heteroatoms might be favored due to the higher hydrophilicity that typically leads to an increased compound solubility. For instance, the aqueous solubility (logS) for cyclopentane, tetrahydrofuran, and pyrrolidine is −2.64, 0.56, and 1.15, respectively. A similar trend is observable with cyclohexane (−3.1), tetrahydropyran (−0.03), and piperidine (1.07) 20. Moreover, the presence of a heteroatom can be functional to the formation of interactions such as hydrogen bonds. Carbo-aliphatic rings, on the other hand, generally do not offer the opportunity of increased potency or improved physicochemical properties. These reasons, in addition to their lipophilic character, might be some of the causes of their underrepresentation in drug molecules and medicinal chemistry compounds. Among frequent 3D fragments, rings coming from natural product structures are common, e.g., *β*-lactams (**16**) and steroid scaffolds, targeting specifically penicillin-binding proteins and nuclear receptors, respectively.

Next, we examined if any fragments are ‘privileged’ in terms of target class. Table 2 lists the highest 16 fragments that are present in at least five drugs and are only associated with a single target class. 15 fragments are 3D ring assemblies, with only one 2D fragment (**34**)**.** Fragment **34** is found in nine drugs targeting bacterial topoisomerases. Note that five fragments (**27**,**46**,**47**,**55**, and **56**) are steroid-type. A few of these structures (e.g., **27** and **55**) might appear to be repeated, as they derive from different stereoisomers. Contrary to the most common 3D fragments listed in Table 1, where 3D-only monocycles are by far the most commonly employed systems, here most of the 3D systems are medium size assemblies, comprising two, three or four rings, and seven of fifteen 3D fragments are 3D hybrid systems. Without careful experimental investigation, it is not possible to determine whether the scaffolds/fragments present in different drugs targeting the same protein not only contribute to the binding affinity but also the specificity. However, such ring systems have been successfully employed to target similar biomolecules, whereas other assemblies have been exploited to target a multiplicity of protein classes, suggesting they might unselectively contribute to binding. Despite being aware of the hypothetical nature of this rationale, these results could corroborate the idea that 3D assemblies may contribute to the specificity of a bioactive compound more effectively than a two-dimensional ring scaffold as suggested by Clemons *et al*. 14. In the context of fragment-based drug design, 3D fragments might thus constitute higher quality hits, offering also, as shown in the previous section, a more diverse chemistry.

The differences recorded for the ring systems targeting different classes of biomolecules suggest that the fragments, like the whole molecules, depend on the nature of the proteins and their binding sites being targeted. When designing fragments for screening, the protein target information and the shape/saturation of the fragments are all quite important to avoid the risk of underrepresenting 3D fragments as compared to 2D aromatic assemblies.

### Ring fragment similarity

**Figure 7:**
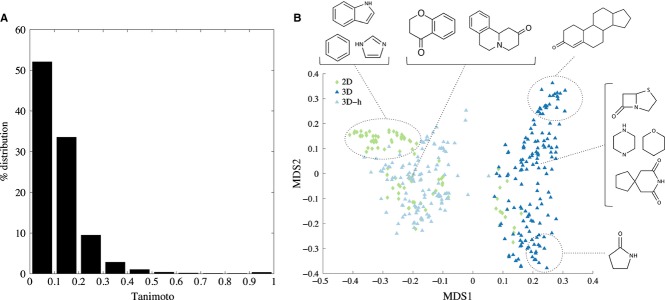
(A) Percentage distribution of Tanimoto scores for the whole set of non-redundant fragments. (B) The first 2 dimensions of the multidimensional scaling (MDS) map of fragments. Three dimensional fragments are denoted by blue triangles and 2D fragments by green diamonds. Some example molecules are shown, representing the structural characteristics of the fragments found in different clusters. Notice the wider spread of 3D fragments on the map, suggesting a higher skeletal diversity.

To investigate the diversity among these rings, the non-redundant fragment structures were compared by first calculating an all-by-all similarity matrix using the Tanimoto coefficient. The distribution of the Tanimoto values is plotted in Figure 7A. The pairwise results showed the most fragments are quite dissimilar (84.6% of fragment pairs have Tanimoto less than 0.2); indicating that globally the fragment set is very diverse.

The first 2 dimensions of the MDS of the set of fragments is shown in Figure 7B, covering 33% of the original distance matrix. Aromatic rings such as benzene, naphthalene, pyridine, pyrazine, imidazole, triazole, and indole are clustered in the top-left corner of the plot. Some of these fragments are among the most frequent ring assemblies found in drug molecules. Below this cluster are 2D rings characterized by the presence of carbonyl groups and hetero-aromatic tri-cyclic ring systems. These rings are found in drugs such as the antipsychotic Enprofylline and the antineoplastic Mitoxantron. The other 2D fragments on the left-hand side of the map are mainly polycyclic. Three dimensional fragments occupying the right-hand side of the plot are highly saturated compounds, containing only aliphatic rings, such as cyclohexane, piperazine, tetrahydropyran, some spiro compounds and the fused beta-lactam structures that are the core of many antibiotics. 3D-h fragments are positioned closer to 2D fragments on the map as they share the same structure of some single rings. The very top-right corner is populated by steroid-like structures. Three dimensional fragments at the bottom-right of the plot are monocycles characterized by the presence of peptide bonds, such as *β*- and *γ*-lactams and cyclic peptides. In general, from this map is possible to notice how 3D ring assemblies scatter more homogeneously on the plot, suggesting a slightly larger structural diversity.

**Figure 8:**
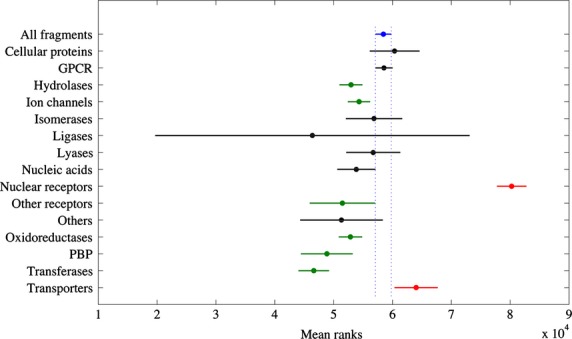
Fragment similarity comparison between different target classes. Each line represents the distribution of Tanimoto scores for the fragments belonging to a specific target class. Shown are the location of the distribution and a 95% confidence interval (in blue dotted box for the all-fragment sample). Two mean ranks are significantly different if their intervals do not overlap. Samples in red show a significantly higher mean rank (lower diversity) from a chosen reference group, representing all the fragments (in blue). Samples in green show a significantly lower mean rank (higher diversity) as compared to the reference.

Next, the fragment similarity within each target class was investigated. Since we defined broad target classes, each class may consist of a number of protein sub-families representing a different degree of biological diversity in terms of proteins’ structures and functions. Although drugs targeting the same protein may not necessarily bind to the same site, fragments related to a small target class might be more biased by the presence of compounds very similar to each other. Thus, in order to determine whether the presence of particular compound classes would substantially influence the outcome of the analysis, the pairwise Tanimoto similarities for the fragments within each target class were compared to those for all fragments. The results (data not shown) showed that the fragments within each target class have high diversity. As a general trend, as seen in Figure 7A, more than 50% of Tanimoto scores in all target classes are below 0.2. Eight classes out of fifteen have at least 90% of their Tanimoto scores below 0.3, including GPCRs, ion channels, oxidoreductases, and transferases. Next, we applied the Kruskal–Wallis one-way test for the Tanimoto scores for each target versus the all-by-all fragments. The results in Figure 8 show that seven target classes (in black) have comparable fragment similarity to the group representing all non-redundant fragments (Figure 7A). On the other hand, six classes (hydrolases, ion channels, oxidoreductases, penicillin-binding proteins, transferases and other receptors, in green) have a similarity distribution shifted toward lower values, indicating higher diversity as compared to the whole set of fragments. The only two classes (in red) showing less diversity than the reference are nuclear receptors and transporters.

The high percentage of 3D fragments in nuclear receptors is in fact influenced by the presence of many steroid-type structures (e.g., **46**,**47**,**55**, and **56** in Table 2). Their fragment structures are unique to each other, although very similar. On the other hand, 3D structures in transporters seem not to be influenced by any particular type of fragment and the class shows a very high degree of fragment diversity with 89.3% of its Tanimoto scores below 0.3. The set of ring systems within the cellular proteins class shows low similarity. However, a closer inspection of the structures reveals that the high proportion of 3D fragments is largely due to the presence of many natural macrocycles. In fact, 10 of the 28 fragments in this category are rings composed of more than eight atoms (e.g., **219**,**244**,**258,** and **262** in Table S1) and are mainly derived from macrolide antibiotics and anti-microtubule agents. Even taking into account the similarity of fragments within nuclear receptors and the presence of many macrocycles within cellular proteins, it is hard to deny the substantial contribution of three-dimensional structures for these two target classes. Overall, for any protein target in Figure 6, the presence of a 3D fragment is quite important in the drug structure. When designing a focused library, or selecting fragments for screening, it may be useful to take into account the target class information as well as structural information. From this analysis, it seems that for some target classes, especially receptors, such as nuclear receptors, transporters and ion channels, 3D rings contribute positively toward good lead compounds.

## Conclusion

We carried out this study to analyze 2D and 3D fragments in drugs as well as characterize their molecular profiles, diversity, and target classes. Our data and analysis provide useful information on the variety of fragments and their properties, with the linking information on specific target classes.

What first emerged from the study is that although flat aromatic scaffolds are widely employed, three-dimensional aliphatic ring fragments offer considerably greater structural and shape diversity. The results support the hypothesis that 3D fragments allow the sampling of a larger chemical space. The presence of so many different 3D structures in marketed drugs and their increased synthetic accessibility is reassuring considering the suggested detrimental properties on drug candidates brought by a high aromatic ring count.

Second, considerable differences in 3D ring presence were recorded among different targets classes, such as nuclear receptors, transporters, ion channels, and GPCRs. It appears that the preference toward specific scaffold shapes is likely to be highly target dependent. This information can be taken into account in the context of fragment library design in order to optimize 3D fragment content, in particular when addressing drug targets poorly amenable to flat compounds. Considering the diverse characteristics of each protein class, the composition of a library should probably be a function of the biological target explored.

Finally, from the perspective of drug design, the appearance of common ring fragments reflects the limited variability of currently available chemical libraries from which drugs are derived. Therefore, the results of this article will also be useful in directing new synthetic chemical efforts toward poorly explored areas of drug-like space.

Research on the effects of 3D rings on the frequency and quality of fragment hits for different targets would contribute to knowledge in this area. Analysis into other data sets (e.g., published medicinal compounds) might provide additional information on the contribution of 3D rings to bioactive compounds and their protein targets.

## Notes

*Pipeline Pilot 8.5*. Pipeline Pilot is avaliable from Accelrys, http://accelrys.com/products/pipeline-pilot.

*Matlab R2012b*. Mathworks. http://www.mathworks.co.uk/.

*DrugPort* EMBL-EBI. http://www.ebi.ac.uk/thornton-srv/databases/drugport.

*Water Solubility (logS) database*. ADME team, University of California, San Diego. http://modem.ucsd.edu/adme/databases/databases_logS.htm.

## Supporting Information

**Table S1** Non-redundant ring fragments in drugs
