# Supplementary material for: Two- and Three-dimensional Rings in Drugs
Source: Chem Biol Drug Des. 2014 Jan 1;83(4):450–61. doi: 10.1111/cbdd.12260 (PMC4233953; doi:10.1111/cbdd.12260)
Supplement: Supplementary file 1 [file cbdd0083-0450-SD1.docx]

| Table S1. Non-redundant ring fragments in drugs | | | | | | |
| --- | --- | --- | --- | --- | --- | --- |
| Fragment structure^a^ | Fragment ID^b^ | No. Rings^c^ | Frequency^d^ | No. Drugs^e^ | Target Classes^f^ | No. Target Classes^g^ |
| 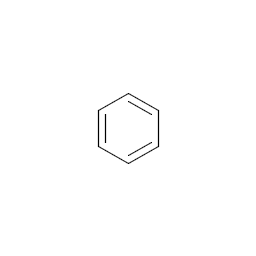 | 1 | 1 | 838 | 616 | Transporters GPCRs Hydrolases Oxidoreductases Ion Channels Other Receptors Lyases Nuclear Receptors Transferases Nucleic acids Penicillin binding proteins Isomerases Cellular proteins Others | 14 |
| 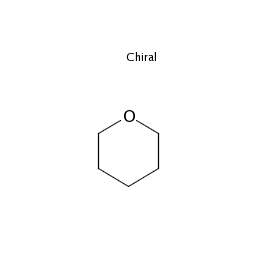 | 2 | 1 | 90 | 48 | Cellular proteins Hydrolases Ligases Isomerases Transporters Transferases Ion Channels Others Nucleic acids | 9 |
| 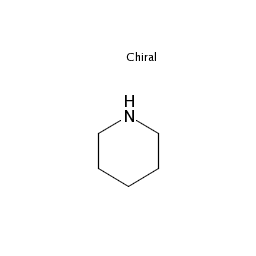 | 3 | 1 | 74 | 70 | Hydrolases Ion Channels GPCRs Oxidoreductases Transferases Transporters Nuclear Receptors Isomerases Other Receptors | 9 |
| 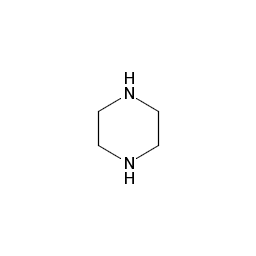 | 4 | 1 | 65 | 64 | Hydrolases Nucleic acids Ion Channels GPCRs Oxidoreductases Isomerases Transferases | 7 |
| 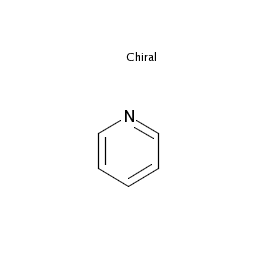 | 5 | 1 | 62 | 60 | Ion Channels Hydrolases Transporters GPCRs Transferases Nuclear Receptors Penicillin binding proteins Oxidoreductases Isomerases Lyases Nucleic acids Cellular proteins | 12 |
| 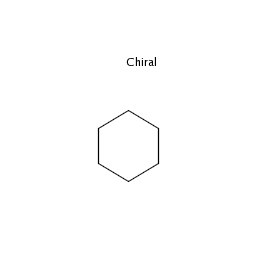 | 6 | 1 | 59 | 57 | GPCRs Hydrolases Other Receptors Transporters Others Isomerases Ion Channels Cellular proteins Nuclear Receptors Penicillin binding proteins Lyases Transferases Oxidoreductases Nucleic acids | 14 |
| 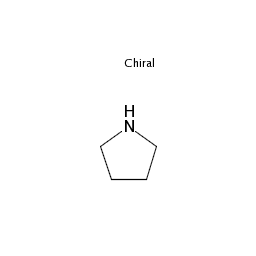 | 7 | 1 | 37 | 35 | Ion Channels GPCRs Penicillin binding proteins Hydrolases Lyases Cellular proteins Transporters | 7 |
| 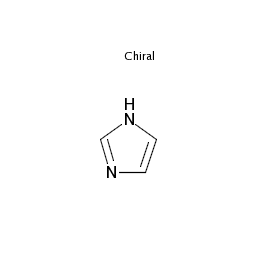 | 8 | 1 | 32 | 32 | Hydrolases Oxidoreductases GPCRs Ligases Lyases Others Nucleic acids Ion Channels Transferases | 9 |
| 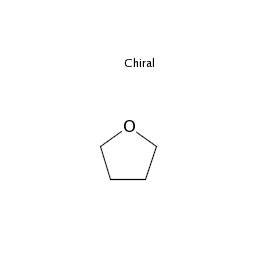 | 9 | 1 | 30 | 30 | Transferases Oxidoreductases Lyases GPCRs Cellular proteins Hydrolases Nucleic acids | 7 |
| 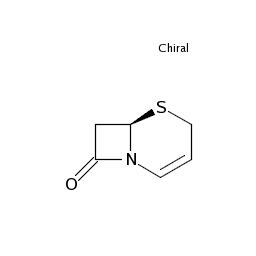 | 10 | 2 | 28 | 28 | Penicillin binding proteins | 1 |
| 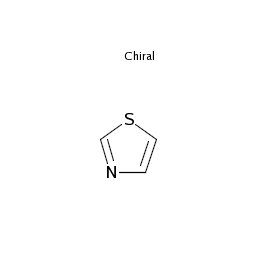 | 11 | 1 | 27 | 24 | Penicillin binding proteins Ligases Hydrolases Oxidoreductases GPCRs Transferases Cellular proteins | 7 |
| 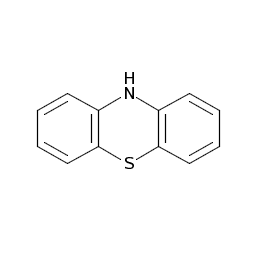 | 12 | 3 | 25 | 25 | GPCRs Ion Channels | 2 |
| 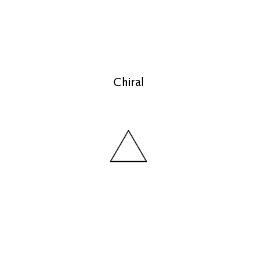 | 13 | 1 | 22 | 22 | GPCRs Hydrolases Isomerases Oxidoreductases Transferases Lyases Ion Channels Transporters | 8 |
| 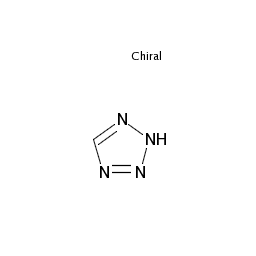 | 14 | 1 | 21 | 21 | GPCRs Penicillin binding proteins Hydrolases | 3 |
| 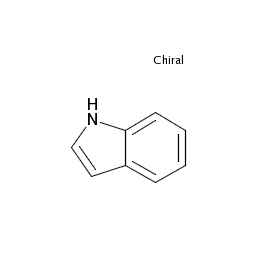 | 15 | 2 | 20 | 19 | Other Receptors GPCRs Oxidoreductases Transferases Ion Channels | 5 |
| 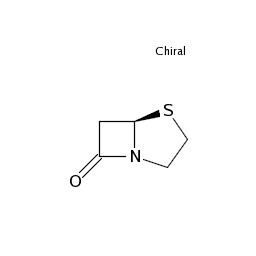 | 16 | 2 | 20 | 20 | Penicillin binding proteins | 1 |
| 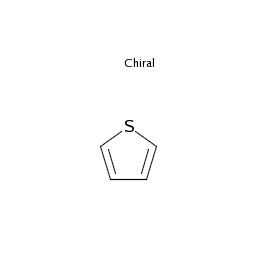 | 17 | 1 | 19 | 17 | Lyases Isomerases Penicillin binding proteins Transporters GPCRs Oxidoreductases | 6 |
| 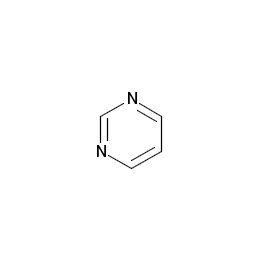 | 18 | 1 | 18 | 17 | Oxidoreductases Ligases Transferases GPCRs Other Receptors | 5 |
| 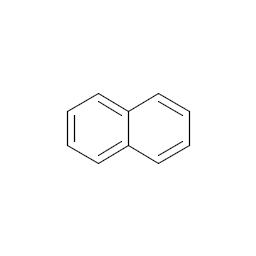 | 19 | 2 | 16 | 15 | Nuclear Receptors Oxidoreductases Transporters GPCRs Penicillin binding proteins Other Receptors | 6 |
| 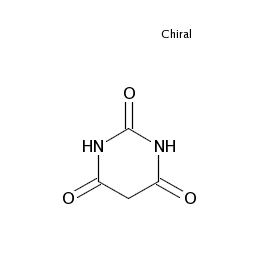 | 20 | 1 | 15 | 15 | Ion Channels | 1 |
| 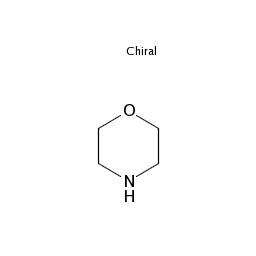 | 21 | 1 | 15 | 15 | Transporters Transferases GPCRs Ion Channels Nucleic acids Oxidoreductases | 6 |
| 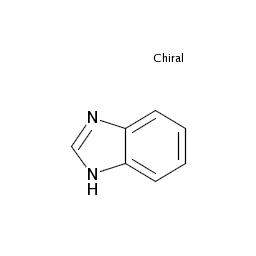 | 22 | 2 | 14 | 13 | Hydrolases Cellular proteins GPCRs Oxidoreductases | 4 |
| 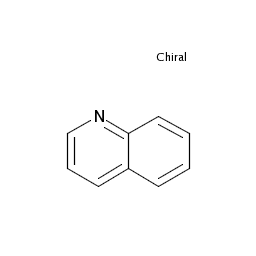 | 23 | 2 | 14 | 14 | GPCRs Others Ion Channels Other Receptors Transferases Hydrolases Oxidoreductases | 7 |
| 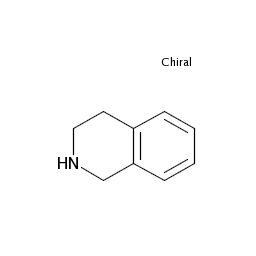 | 24 | 2 | 14 | 10 | Ion Channels Hydrolases GPCRs Transporters | 4 |
| 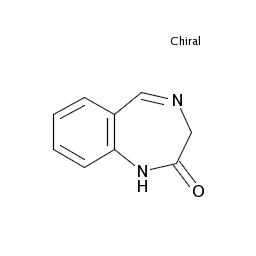 | 25 | 2 | 14 | 14 | Ion Channels | 1 |
| 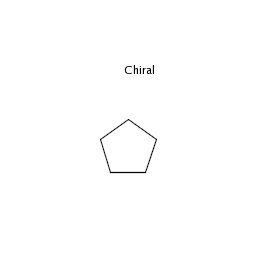 | 26 | 1 | 13 | 13 | GPCRs Hydrolases Transferases Nuclear Receptors | 4 |
| 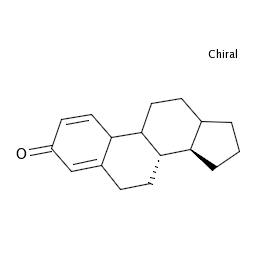 | 27 | 4 | 12 | 12 | Nuclear Receptors | 1 |
| 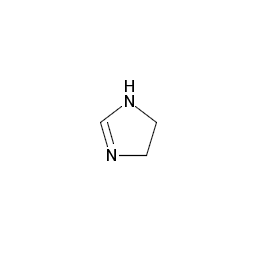 | 28 | 1 | 12 | 12 | GPCRs | 1 |
| 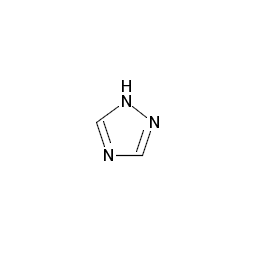 | 29 | 1 | 11 | 10 | Oxidoreductases GPCRs | 2 |
| 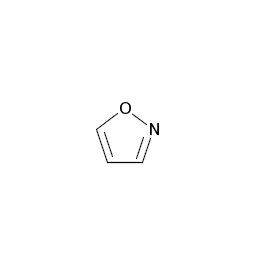 | 30 | 1 | 11 | 11 | Transferases Penicillin binding proteins Oxidoreductases Other Receptors GPCRs | 5 |
| 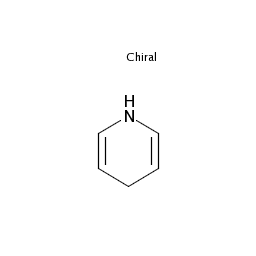 | 31 | 1 | 10 | 10 | Ion Channels Nuclear Receptors GPCRs | 3 |
| 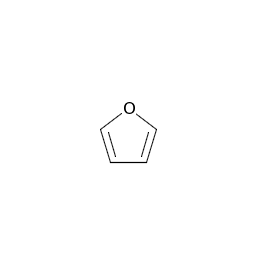 | 32 | 1 | 10 | 10 | Oxidoreductases GPCRs Nucleic acids Lyases Penicillin binding proteins Other Receptors Transferases | 7 |
| 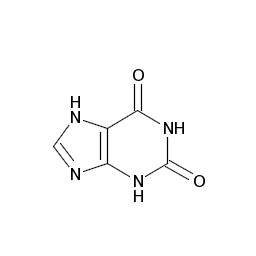 | 33 | 2 | 10 | 9 | GPCRs Hydrolases | 2 |
| 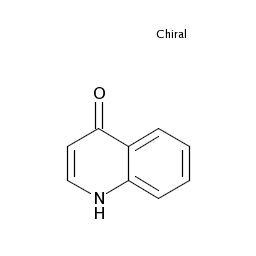 | 34 | 2 | 9 | 9 | Isomerases | 1 |
| 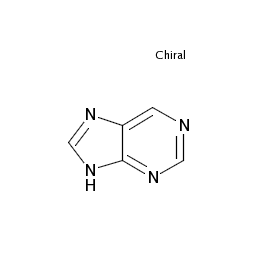 | 35 | 2 | 9 | 9 | Transferases Oxidoreductases Nucleic acids GPCRs | 4 |
| 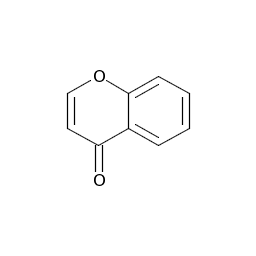 | 36 | 2 | 9 | 7 | Oxidoreductases Ion Channels GPCRs | 3 |
| 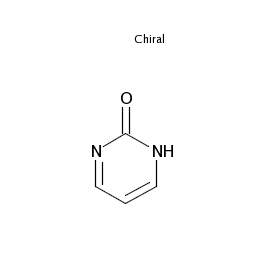 | 37 | 1 | 9 | 9 | Transferases Lyases | 2 |
| 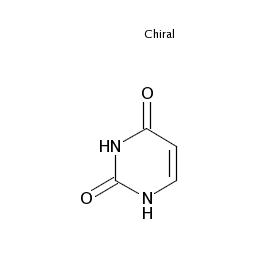 | 38 | 1 | 8 | 8 | Transferases Lyases Nucleic acids | 3 |
| 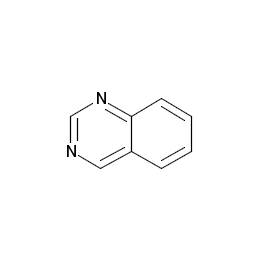 | 39 | 2 | 8 | 8 | Transferases GPCRs Oxidoreductases | 3 |
| 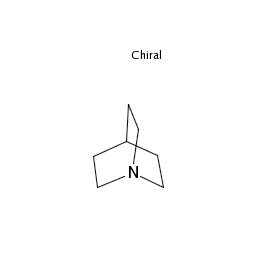 | 40 | 2 | 8 | 8 | Ion Channels Others GPCRs Cellular proteins | 4 |
| 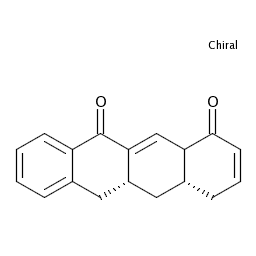 | 41 | 4 | 7 | 7 | Cellular proteins | 1 |
| 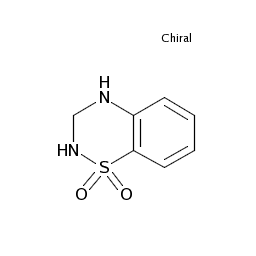 | 42 | 2 | 7 | 7 | Lyases Transporters Hydrolases | 3 |
| 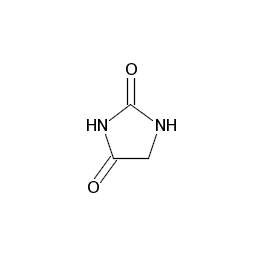 | 43 | 1 | 7 | 7 | Ion Channels Nuclear Receptors Oxidoreductases Other Receptors | 4 |
| 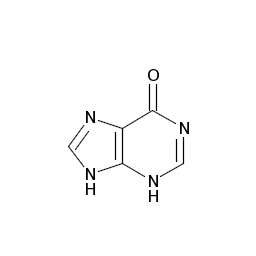 | 44 | 2 | 7 | 7 | Transferases Nucleic acids | 2 |
| 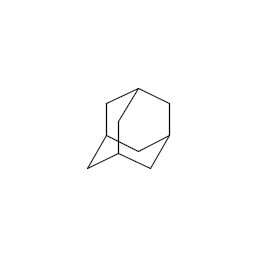 | 45 | 3 | 6 | 6 | Nuclear Receptors Others GPCRs Ion Channels Hydrolases | 5 |
| 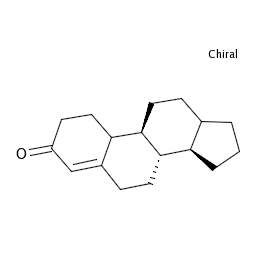 | 46 | 4 | 6 | 6 | Nuclear Receptors | 1 |
| 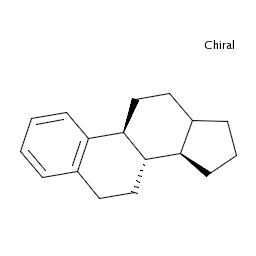 | 47 | 4 | 6 | 6 | Nuclear Receptors | 1 |
| 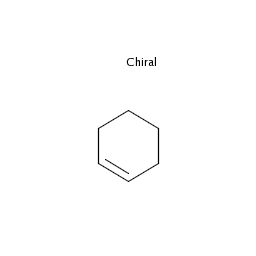 | 48 | 1 | 6 | 6 | Hydrolases Nuclear Receptors Ion Channels GPCRs | 4 |
| 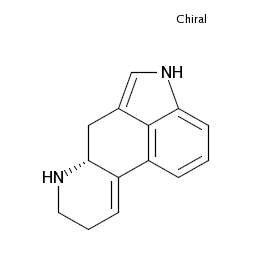 | 49 | 4 | 6 | 6 | GPCRs | 1 |
| 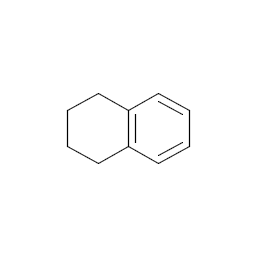 | 50 | 2 | 5 | 5 | Nuclear Receptors Transporters GPCRs | 3 |
| 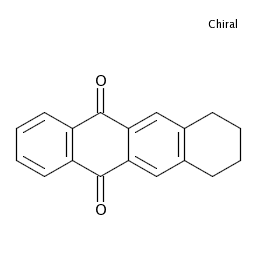 | 51 | 4 | 5 | 5 | Isomerases | 1 |
| 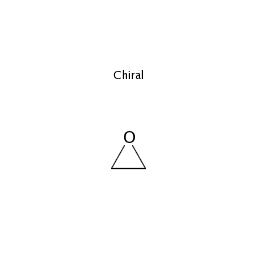 | 52 | 1 | 5 | 5 | Ligases Transferases | 2 |
| 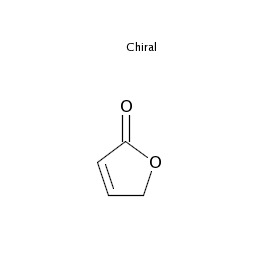 | 53 | 1 | 5 | 5 | Hydrolases | 1 |
| 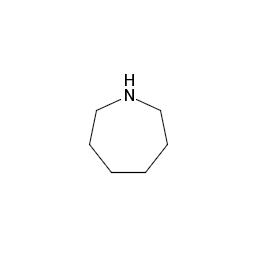 | 54 | 1 | 5 | 5 | Other Receptors GPCRs Penicillin binding proteins | 3 |
| 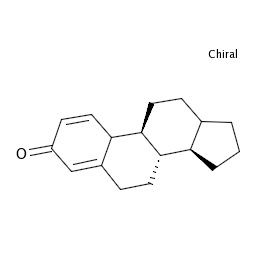 | 55 | 4 | 5 | 5 | Nuclear Receptors | 1 |
| 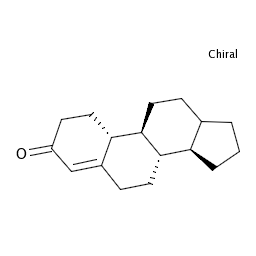 | 56 | 4 | 5 | 5 | Nuclear Receptors | 1 |
| 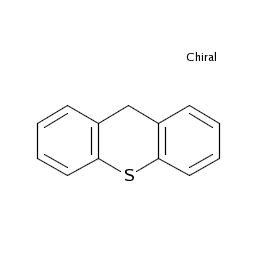 | 57 | 3 | 5 | 5 | GPCRs | 1 |
| 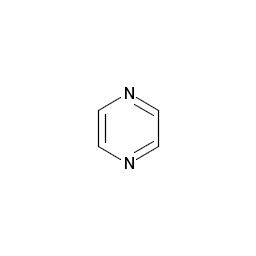 | 58 | 1 | 5 | 5 | Ion Channels Transferases Other Receptors Hydrolases | 4 |
| 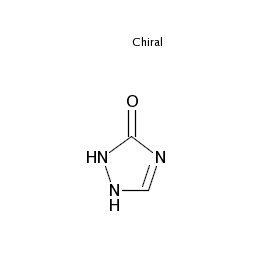 | 59 | 1 | 5 | 5 | GPCRs Oxidoreductases | 2 |
| 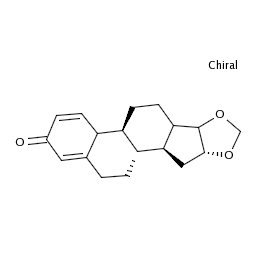 | 60 | 5 | 4 | 4 | Nuclear Receptors | 1 |
| 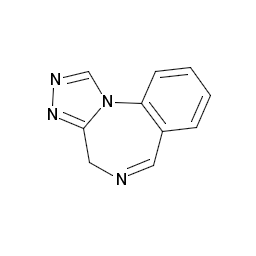 | 61 | 3 | 4 | 4 | Ion Channels | 1 |
| 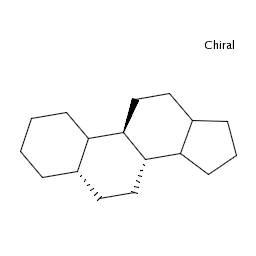 | 62 | 4 | 4 | 4 | Hydrolases | 1 |
| 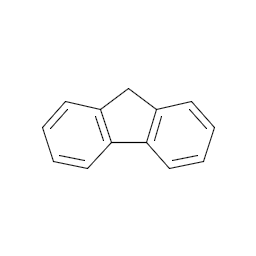 | 63 | 3 | 4 | 3 | Ion Channels Hydrolases | 2 |
| 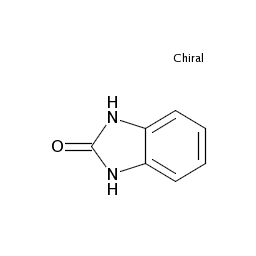 | 64 | 2 | 4 | 3 | GPCRs | 1 |
| 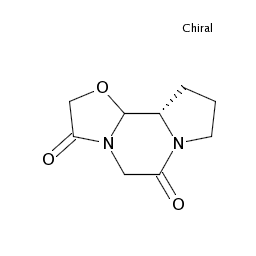 | 65 | 3 | 4 | 4 | GPCRs | 1 |
| 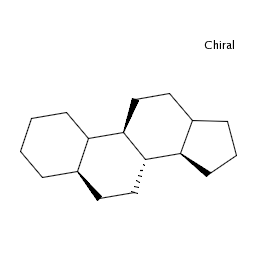 | 66 | 4 | 4 | 4 | GPCRs Ion Channels | 2 |
| 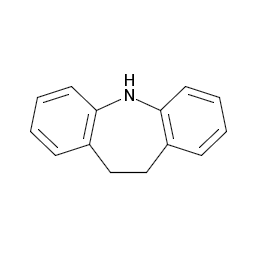 | 67 | 3 | 4 | 4 | GPCRs Transporters | 2 |
| 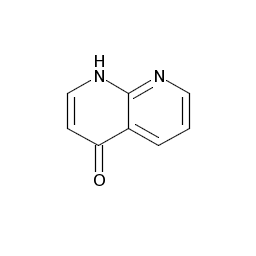 | 68 | 2 | 4 | 4 | Isomerases Nucleic acids Lyases | 3 |
| 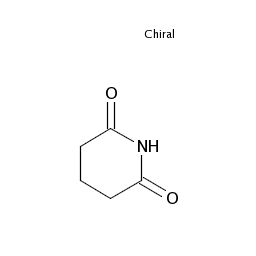 | 69 | 1 | 4 | 4 | Oxidoreductases Ion Channels | 2 |
| 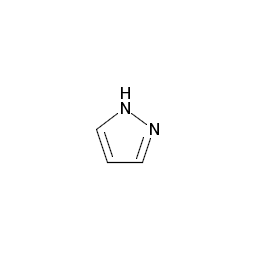 | 70 | 1 | 4 | 4 | GPCRs Oxidoreductases | 2 |
| 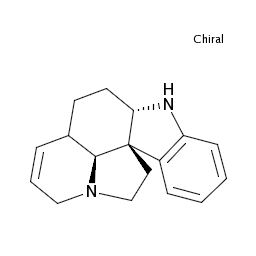 | 71 | 5 | 4 | 4 | Cellular proteins | 1 |
| 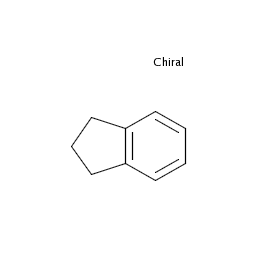 | 72 | 2 | 4 | 4 | Hydrolases Oxidoreductases Ion Channels | 3 |
| 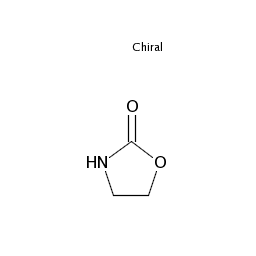 | 73 | 1 | 4 | 4 | GPCRs Nucleic acids | 2 |
| 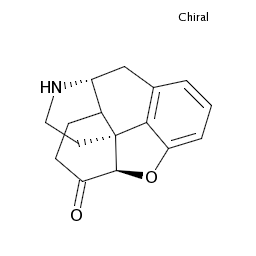 | 74 | 5 | 4 | 4 | GPCRs | 1 |
| 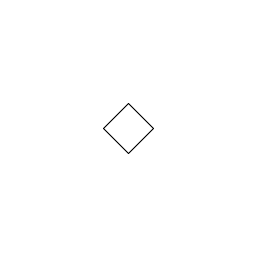 | 75 | 1 | 4 | 4 | GPCRs Transporters | 2 |
| 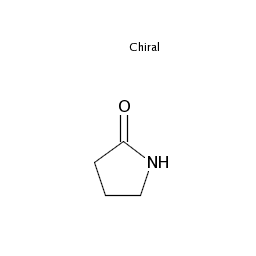 | 76 | 1 | 4 | 4 | Ion Channels GPCRs Transporters | 3 |
| 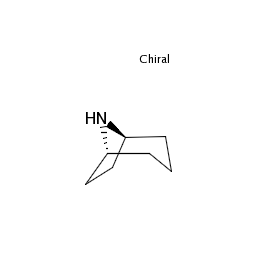 | 77 | 2 | 4 | 4 | GPCRs Cellular proteins | 2 |
| 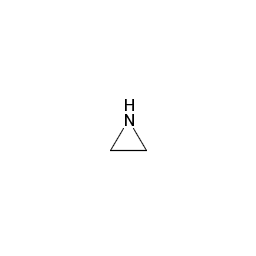 | 78 | 1 | 3 | 1 | Nucleic acids | 1 |
| 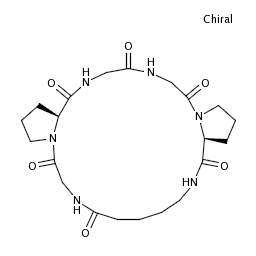 | 79 | 3 | 3 | 3 | Transferases | 1 |
| 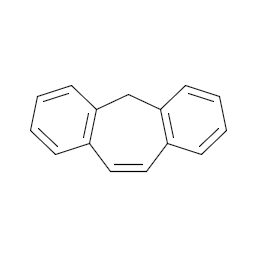 | 80 | 3 | 3 | 3 | Transporters GPCRs | 2 |
| 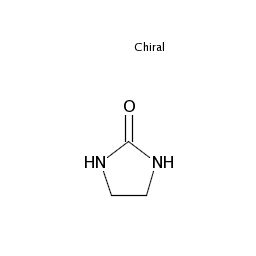 | 81 | 1 | 3 | 3 | Penicillin binding proteins GPCRs | 2 |
| 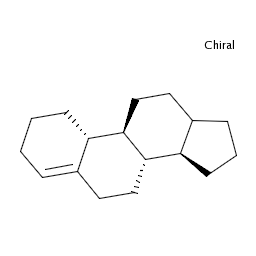 | 82 | 4 | 3 | 3 | Nuclear Receptors | 1 |
| 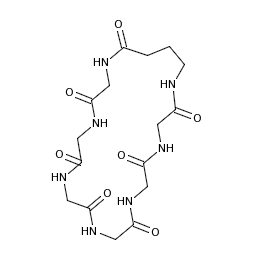 | 83 | 1 | 3 | 3 |  |  |
| 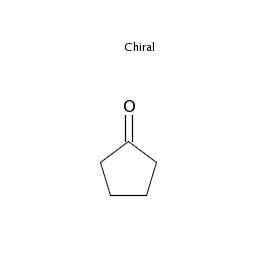 | 84 | 1 | 3 | 3 | GPCRs | 1 |
| 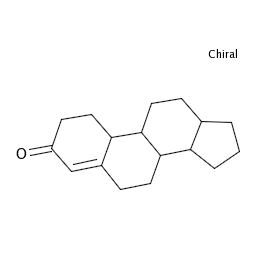 | 85 | 4 | 3 | 3 | Nuclear Receptors | 1 |
| 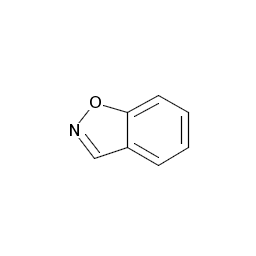 | 86 | 2 | 3 | 3 | GPCRs Ion Channels | 2 |
| 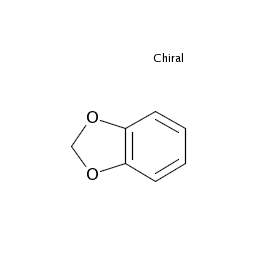 | 87 | 2 | 3 | 3 | Transporters Hydrolases GPCRs | 3 |
| 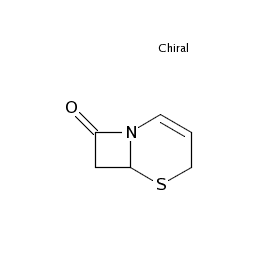 | 88 | 2 | 3 | 3 | Penicillin binding proteins | 1 |
| 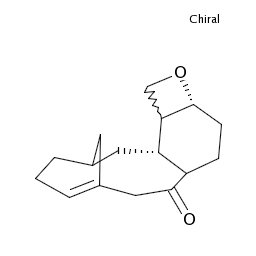 | 89 | 4 | 3 | 3 | Cellular proteins | 1 |
| 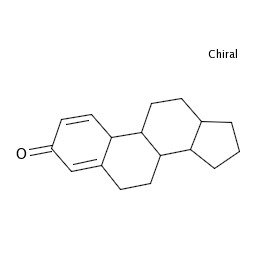 | 90 | 4 | 3 | 3 | Nuclear Receptors | 1 |
| 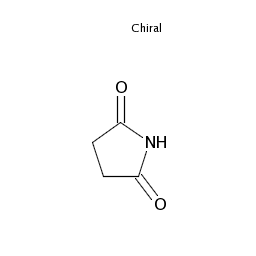 | 91 | 1 | 3 | 3 | Ion Channels | 1 |
| 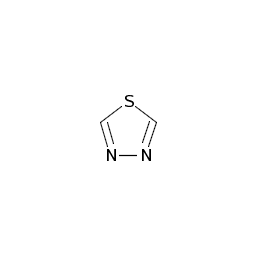 | 92 | 1 | 3 | 3 | Transferases Lyases Penicillin binding proteins | 3 |
| 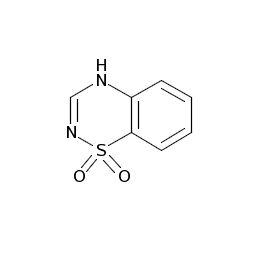 | 93 | 2 | 3 | 3 | Lyases Hydrolases | 2 |
| 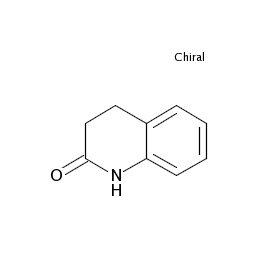 | 94 | 2 | 3 | 3 | GPCRs Hydrolases | 2 |
| 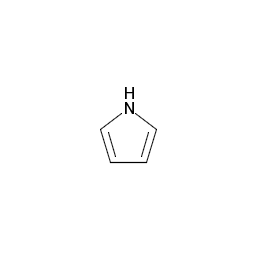 | 95 | 1 | 3 | 3 | Oxidoreductases Transferases | 2 |
| 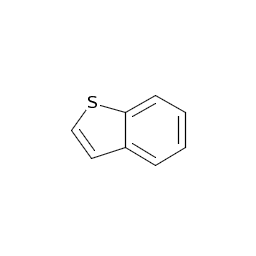 | 96 | 2 | 3 | 3 | Nuclear Receptors Oxidoreductases | 2 |
| 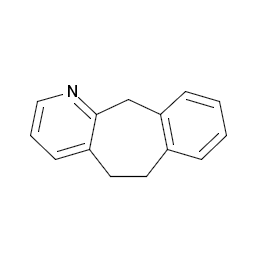 | 97 | 3 | 3 | 3 | GPCRs | 1 |
| 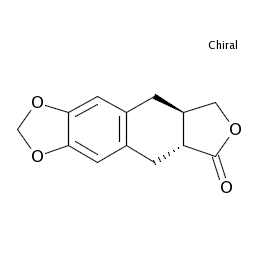 | 98 | 4 | 3 | 3 | Isomerases | 1 |
| 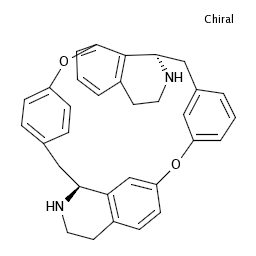 | 99 | 7 | 3 | 3 | Ion Channels GPCRs | 2 |
| 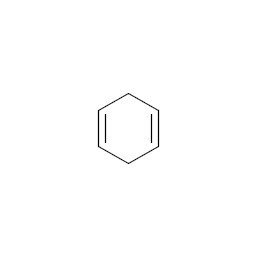 | 100 | 1 | 3 | 3 | Nucleic acids Penicillin binding proteins | 2 |
|  | 101 | 5 | 3 | 3 | Transporters Hydrolases | 2 |
|  | 102 | 4 | 3 | 3 | Cellular proteins | 1 |
|  | 103 | 1 | 3 | 2 | Nucleic acids GPCRs | 2 |
|  | 104 | 1 | 3 | 3 | Hydrolases | 1 |
|  | 105 | 2 | 3 | 3 | GPCRs Transferases | 2 |
|  | 106 | 3 | 3 | 3 | Nucleic acids Hydrolases Others | 3 |
|  | 107 | 4 | 3 | 3 | GPCRs | 1 |
|  | 108 | 1 | 3 | 3 | Oxidoreductases | 1 |
|  | 109 | 2 | 3 | 3 | Oxidoreductases | 1 |
|  | 110 | 2 | 3 | 3 | Penicillin binding proteins | 1 |
|  | 111 | 2 | 2 | 2 | Oxidoreductases | 1 |
|  | 112 | 4 | 2 | 2 | Nuclear Receptors | 1 |
|  | 113 | 2 | 2 | 2 | Ion Channels Transporters | 2 |
|  | 114 | 4 | 2 | 2 | Nuclear Receptors | 1 |
|  | 115 | 4 | 2 | 2 | GPCRs | 1 |
|  | 116 | 4 | 2 | 2 | Cellular proteins Oxidoreductases | 2 |
|  | 117 | 5 | 2 | 1 | Other Receptors | 1 |
|  | 118 | 2 | 2 | 2 | Lyases GPCRs | 2 |
|  | 119 | 2 | 2 | 2 | Oxidoreductases GPCRs | 2 |
|  | 120 | 5 | 2 | 1 | Ion Channels | 1 |
|  | 121 | 1 | 2 | 2 | Ion Channels | 1 |
|  | 122 | 1 | 2 | 2 | Oxidoreductases | 1 |
|  | 123 | 5 | 2 | 2 | Nuclear Receptors | 1 |
|  | 124 | 2 | 2 | 2 | GPCRs | 1 |
|  | 125 | 1 | 2 | 2 | Hydrolases Cellular proteins | 2 |
|  | 126 | 3 | 2 | 2 | Nucleic acids | 1 |
|  | 127 | 1 | 2 | 2 | Nucleic acids | 1 |
|  | 128 | 2 | 2 | 2 | Transporters Lyases | 2 |
|  | 129 | 4 | 2 | 2 | GPCRs | 1 |
|  | 130 | 2 | 2 | 2 | Oxidoreductases Lyases | 2 |
|  | 131 | 1 | 2 | 2 | Nucleic acids Hydrolases | 2 |
|  | 132 | 1 | 2 | 2 | Nucleic acids | 1 |
|  | 133 | 2 | 2 | 1 | Nucleic acids | 1 |
|  | 134 | 2 | 2 | 2 | Lyases Transferases | 2 |
|  | 135 | 3 | 2 | 2 | Isomerases Transferases | 2 |
|  | 136 | 1 | 2 | 2 | Oxidoreductases Transferases | 2 |
|  | 137 | 2 | 2 | 2 | Transferases | 1 |
|  | 138 | 2 | 2 | 2 | Lyases Oxidoreductases | 2 |
|  | 139 | 3 | 2 | 2 | GPCRs | 1 |
|  | 140 | 1 | 2 | 2 | Ion Channels Nucleic acids | 2 |
|  | 141 | 3 | 2 | 2 | Oxidoreductases GPCRs | 2 |
|  | 142 | 4 | 2 | 2 | Transferases | 1 |
|  | 143 | 1 | 2 | 2 | Oxidoreductases Transporters | 2 |
|  | 144 | 2 | 2 | 2 | Hydrolases GPCRs | 2 |
|  | 145 | 1 | 2 | 2 | Cellular proteins | 1 |
|  | 146 | 3 | 2 | 2 | GPCRs | 1 |
|  | 147 | 2 | 2 | 2 | Hydrolases | 1 |
|  | 148 | 3 | 2 | 2 | GPCRs | 1 |
|  | 149 | 5 | 2 | 2 | Isomerases | 1 |
|  | 150 | 2 | 2 | 2 | GPCRs | 1 |
|  | 151 | 2 | 2 | 2 | GPCRs | 1 |
|  | 152 | 2 | 2 | 2 | GPCRs | 1 |
|  | 153 | 1 | 2 | 1 | GPCRs | 1 |
|  | 154 | 2 | 2 | 2 | Transporters GPCRs | 2 |
|  | 155 | 2 | 2 | 2 | Oxidoreductases | 1 |
|  | 156 | 3 | 2 | 2 | Isomerases | 1 |
|  | 157 | 1 | 2 | 2 | Transferases | 1 |
|  | 158 | 1 | 2 | 2 | Oxidoreductases | 1 |
|  | 159 | 5 | 2 | 2 | GPCRs | 1 |
|  | 160 | 3 | 2 | 2 | GPCRs | 1 |
|  | 161 | 2 | 2 | 2 | Isomerases | 1 |
|  | 162 | 2 | 2 | 2 | Oxidoreductases Lyases | 2 |
|  | 163 | 1 | 2 | 2 | GPCRs | 1 |
|  | 164 | 1 | 2 | 2 | Penicillin binding proteins Transferases | 2 |
|  | 165 | 4 | 2 | 2 | Oxidoreductases Nuclear Receptors | 2 |
|  | 166 | 2 | 2 | 2 | GPCRs Transporters | 2 |
|  | 167 | 1 | 2 | 1 | Nucleic acids | 1 |
|  | 168 | 2 | 2 | 2 | Lyases Ion Channels | 2 |
|  | 169 | 1 | 2 | 2 | GPCRs | 1 |
|  | 170 | 1 | 2 | 2 | Nuclear Receptors | 1 |
|  | 171 | 3 | 2 | 2 | GPCRs | 1 |
|  | 172 | 2 | 2 | 2 | Transporters Oxidoreductases | 2 |
|  | 173 | 5 | 2 | 2 | GPCRs | 1 |
|  | 174 | 1 | 2 | 1 | Isomerases | 1 |
|  | 175 | 2 | 2 | 2 | Ion Channels | 1 |
|  | 176 | 2 | 2 | 2 | Ion Channels Oxidoreductases | 2 |
|  | 177 | 1 | 2 | 2 | Oxidoreductases | 1 |
|  | 178 | 2 | 2 | 1 | GPCRs | 1 |
|  | 179 | 2 | 2 | 2 | Hydrolases Nucleic acids | 2 |
|  | 180 | 1 | 2 | 2 | Penicillin binding proteins Others | 2 |
|  | 181 | 2 | 2 | 2 | Nucleic acids Transferases | 2 |
|  | 182 | 1 | 2 | 2 | Ion Channels | 1 |
|  | 183 | 1 | 1 | 1 | GPCRs | 1 |
|  | 184 | 2 | 1 | 1 | GPCRs | 1 |
|  | 185 | 3 | 1 | 1 | Nucleic acids | 1 |
|  | 186 | 5 | 1 | 1 | Nuclear Receptors | 1 |
|  | 187 | 1 | 1 | 1 | GPCRs | 1 |
|  | 188 | 2 | 1 | 1 | Hydrolases | 1 |
|  | 189 | 2 | 1 | 1 | Hydrolases | 1 |
|  | 190 | 4 | 1 | 1 | Hydrolases | 1 |
|  | 191 | 3 | 1 | 1 | Ion Channels | 1 |
|  | 192 | 2 | 1 | 1 | Ion Channels | 1 |
|  | 193 | 2 | 1 | 1 | Transferases | 1 |
|  | 194 | 1 | 1 | 1 | Ion Channels | 1 |
|  | 195 | 5 | 1 | 1 | Oxidoreductases | 1 |
|  | 196 | 2 | 1 | 1 | Hydrolases | 1 |
|  | 197 | 3 | 1 | 1 | Ion Channels | 1 |
|  | 198 | 2 | 1 | 1 | Nucleic acids | 1 |
|  | 199 | 2 | 1 | 1 | Hydrolases | 1 |
|  | 200 | 1 | 1 | 1 | Hydrolases | 1 |
|  | 201 | 4 | 1 | 1 | GPCRs | 1 |
|  | 202 | 1 | 1 | 1 | Hydrolases | 1 |
|  | 203 | 1 | 1 | 1 | Hydrolases | 1 |
|  | 204 | 2 | 1 | 1 | Other Receptors | 1 |
|  | 205 | 4 | 1 | 1 | Oxidoreductases | 1 |
|  | 206 | 4 | 1 | 1 | Nucleic acids | 1 |
|  | 207 | 2 | 1 | 1 | GPCRs | 1 |
|  | 208 | 3 | 1 | 1 | GPCRs | 1 |
|  | 209 | 1 | 1 | 1 | Oxidoreductases | 1 |
|  | 210 | 4 | 1 | 1 | Transferases | 1 |
|  | 211 | 3 | 1 | 1 | Others | 1 |
|  | 212 | 2 | 1 | 1 | GPCRs | 1 |
|  | 213 | 2 | 1 | 1 | Oxidoreductases | 1 |
|  | 214 | 4 | 1 | 1 | Oxidoreductases | 1 |
|  | 215 | 2 | 1 | 1 | Penicillin binding proteins | 1 |
|  | 216 | 3 | 1 | 1 | Hydrolases | 1 |
|  | 217 | 3 | 1 | 1 | GPCRs | 1 |
|  | 218 | 2 | 1 | 1 | Transporters | 1 |
|  | 219 | 2 | 1 | 1 | Cellular proteins | 1 |
|  | 220 | 1 | 1 | 1 | Transferases | 1 |
|  | 221 | 4 | 1 | 1 | GPCRs | 1 |
|  | 222 | 2 | 1 | 1 | Nucleic acids | 1 |
|  | 223 | 3 | 1 | 1 | GPCRs | 1 |
|  | 224 | 2 | 1 | 1 | Hydrolases | 1 |
|  | 225 | 2 | 1 | 1 | GPCRs | 1 |
|  | 226 | 2 | 1 | 1 | Isomerases | 1 |
|  | 227 | 3 | 1 | 1 |  |  |
|  | 228 | 2 | 1 | 1 | Nuclear Receptors | 1 |
|  | 229 | 3 | 1 | 1 | Transferases | 1 |
|  | 230 | 2 | 1 | 1 | Penicillin binding proteins | 1 |
|  | 231 | 3 | 1 | 1 | GPCRs | 1 |
|  | 232 | 3 | 1 | 1 | Transporters | 1 |
|  | 233 | 1 | 1 | 1 | Hydrolases | 1 |
|  | 234 | 2 | 1 | 1 | Hydrolases | 1 |
|  | 235 | 6 | 1 | 1 | Ion Channels | 1 |
|  | 236 | 4 | 1 | 1 |  |  |
|  | 237 | 2 | 1 | 1 | Hydrolases | 1 |
|  | 238 | 1 | 1 | 1 | Hydrolases | 1 |
|  | 239 | 2 | 1 | 1 | GPCRs | 1 |
|  | 240 | 4 | 1 | 1 | Ion Channels | 1 |
|  | 241 | 2 | 1 | 1 | Oxidoreductases | 1 |
|  | 242 | 3 | 1 | 1 | Isomerases | 1 |
|  | 243 | 1 | 1 | 1 | GPCRs | 1 |
|  | 244 | 1 | 1 | 1 | Cellular proteins | 1 |
|  | 245 | 1 | 1 | 1 | Penicillin binding proteins | 1 |
|  | 246 | 2 | 1 | 1 | Hydrolases | 1 |
|  | 247 | 1 | 1 | 1 | Penicillin binding proteins | 1 |
|  | 248 | 3 | 1 | 1 | GPCRs | 1 |
|  | 249 | 2 | 1 | 1 | Hydrolases | 1 |
|  | 250 | 2 | 1 | 1 | Hydrolases | 1 |
|  | 251 | 5 | 1 | 1 | Nuclear Receptors | 1 |
|  | 252 | 7 | 1 | 1 | Nuclear Receptors | 1 |
|  | 253 | 3 | 1 | 1 | Cellular proteins | 1 |
|  | 254 | 4 | 1 | 1 | GPCRs | 1 |
|  | 255 | 2 | 1 | 1 | GPCRs | 1 |
|  | 256 | 1 | 1 | 1 | Ion Channels | 1 |
|  | 257 | 5 | 1 | 1 | GPCRs | 1 |
|  | 258 | 2 | 1 | 1 | Cellular proteins | 1 |
|  | 259 | 2 | 1 | 1 | Hydrolases | 1 |
|  | 260 | 2 | 1 | 1 | GPCRs | 1 |
|  | 261 | 2 | 1 | 1 | Hydrolases | 1 |
|  | 262 | 3 | 1 | 1 | Cellular proteins | 1 |
|  | 263 | 4 | 1 | 1 | Nuclear Receptors | 1 |
|  | 264 | 3 | 1 | 1 | Oxidoreductases | 1 |
|  | 265 | 9 | 1 | 1 | Nucleic acids | 1 |
|  | 266 | 4 | 1 | 1 | Transporters | 1 |
|  | 267 | 3 | 1 | 1 | Transferases | 1 |
|  | 268 | 3 | 1 | 1 | GPCRs | 1 |
|  | 269 | 2 | 1 | 1 | Others | 1 |
|  | 270 | 1 | 1 | 1 | Transporters | 1 |
|  | 271 | 1 | 1 | 1 | Others | 1 |
|  | 272 | 5 | 1 | 1 | Transporters | 1 |
|  | 273 | 2 | 1 | 1 | Lyases | 1 |
|  | 274 | 4 | 1 | 1 | Nuclear Receptors | 1 |
|  | 275 | 3 | 1 | 1 | Isomerases | 1 |
|  | 276 | 3 | 1 | 1 | Ion Channels | 1 |
|  | 277 | 4 | 1 | 1 |  |  |
|  | 278 | 3 | 1 | 1 | GPCRs | 1 |
|  | 279 | 2 | 1 | 1 | GPCRs | 1 |
|  | 280 | 1 | 1 | 1 | Penicillin binding proteins | 1 |
|  | 281 | 4 | 1 | 1 | Oxidoreductases | 1 |
|  | 282 | 3 | 1 | 1 | Ion Channels | 1 |
|  | 283 | 6 | 1 | 1 | Transferases | 1 |
|  | 284 | 1 | 1 | 1 | GPCRs | 1 |
|  | 285 | 3 | 1 | 1 | GPCRs | 1 |
|  | 286 | 5 | 1 | 1 |  |  |
|  | 287 | 2 | 1 | 1 | Hydrolases | 1 |
|  | 288 | 2 | 1 | 1 | GPCRs | 1 |
|  | 289 | 2 | 1 | 1 | Ion Channels | 1 |
|  | 290 | 2 | 1 | 1 | Other Receptors | 1 |
|  | 291 | 2 | 1 | 1 | Other Receptors | 1 |
|  | 292 | 1 | 1 | 1 | Hydrolases | 1 |
|  | 293 | 3 | 1 | 1 | Ion Channels | 1 |
|  | 294 | 4 | 1 | 1 | Nuclear Receptors | 1 |
|  | 295 | 2 | 1 | 1 | Ion Channels | 1 |
|  | 296 | 3 | 1 | 1 | Cellular proteins | 1 |
|  | 297 | 1 | 1 | 1 | GPCRs | 1 |
|  | 298 | 5 | 1 | 1 | Ion Channels | 1 |
|  | 299 | 5 | 1 | 1 | Ion Channels | 1 |
|  | 300 | 2 | 1 | 1 | Oxidoreductases | 1 |
|  | 301 | 3 | 1 | 1 | GPCRs | 1 |
|  | 302 | 6 | 1 | 1 |  |  |
|  | 303 | 1 | 1 | 1 | GPCRs | 1 |
|  | 304 | 2 | 1 | 1 | Penicillin binding proteins | 1 |
|  | 305 | 1 | 1 | 1 | Nucleic acids | 1 |
|  | 306 | 2 | 1 | 1 | Oxidoreductases | 1 |
|  | 307 | 2 | 1 | 1 | Ion Channels | 1 |
|  | 308 | 5 | 1 | 1 | Nuclear Receptors | 1 |
|  | 309 | 2 | 1 | 1 | GPCRs | 1 |
|  | 310 | 3 | 1 | 1 | Cellular proteins | 1 |
|  | 311 | 3 | 1 | 1 | Hydrolases | 1 |
|  | 312 | 4 | 1 | 1 | Nuclear Receptors | 1 |
|  | 313 | 3 | 1 | 1 | Ion Channels | 1 |
|  | 314 | 3 | 1 | 1 | GPCRs | 1 |
|  | 315 | 2 | 1 | 1 | GPCRs | 1 |
|  | 316 | 3 | 1 | 1 | Transporters | 1 |
|  | 317 | 3 | 1 | 1 | Ion Channels | 1 |
|  | 318 | 1 | 1 | 1 | Ion Channels | 1 |
|  | 319 | 2 | 1 | 1 | Oxidoreductases | 1 |
|  | 320 | 2 | 1 | 1 | Hydrolases | 1 |
|  | 321 | 1 | 1 | 1 | Oxidoreductases | 1 |
|  | 322 | 2 | 1 | 1 | Hydrolases | 1 |
|  | 323 | 2 | 1 | 1 | Hydrolases | 1 |
|  | 324 | 4 | 1 | 1 | Other Receptors | 1 |
|  | 325 | 8 | 1 | 1 | Transferases | 1 |
|  | 326 | 2 | 1 | 1 | GPCRs | 1 |
|  | 327 | 2 | 1 | 1 | GPCRs | 1 |
|  | 328 | 3 | 1 | 1 | GPCRs | 1 |
|  | 329 | 2 | 1 | 1 | GPCRs | 1 |
|  | 330 | 2 | 1 | 1 | Ion Channels | 1 |
|  | 331 | 3 | 1 | 1 | GPCRs | 1 |
|  | 332 | 1 | 1 | 1 | GPCRs | 1 |
|  | 333 | 3 | 1 | 1 | Ion Channels | 1 |
|  | 334 | 2 | 1 | 1 | Ion Channels | 1 |
|  | 335 | 3 | 1 | 1 | Hydrolases | 1 |
|  | 336 | 1 | 1 | 1 | Ligases | 1 |
|  | 337 | 2 | 1 | 1 | GPCRs | 1 |
|  | 338 | 3 | 1 | 1 | Oxidoreductases | 1 |
|  | 339 | 2 | 1 | 1 | GPCRs | 1 |
|  | 340 | 2 | 1 | 1 | Transporters | 1 |
|  | 341 | 1 | 1 | 1 | Other Receptors | 1 |
|  | 342 | 2 | 1 | 1 | Hydrolases | 1 |
|  | 343 | 2 | 1 | 1 | Isomerases | 1 |
|  | 344 | 3 | 1 | 1 | GPCRs | 1 |
|  | 345 | 1 | 1 | 1 | Cellular proteins | 1 |
|  | 346 | 3 | 1 | 1 | Nucleic acids | 1 |
|  | 347 | 3 | 1 | 1 | GPCRs | 1 |
|  | 348 | 2 | 1 | 1 | Hydrolases | 1 |
|  | 349 | 1 | 1 | 1 | GPCRs | 1 |
|  | 350 | 4 | 1 | 1 | GPCRs | 1 |
|  | 351 | 3 | 1 | 1 | GPCRs | 1 |
|  | 352 | 4 | 1 | 1 | Cellular proteins | 1 |
|  | 353 | 2 | 1 | 1 | Ion Channels | 1 |
|  | 354 | 4 | 1 | 1 | Nuclear Receptors | 1 |
|  | 355 | 2 | 1 | 1 | Ion Channels | 1 |
|  | 356 | 1 | 1 | 1 | Oxidoreductases | 1 |
|  | 357 | 2 | 1 | 1 | Ion Channels | 1 |
|  | 358 | 3 | 1 | 1 | GPCRs | 1 |
|  | 359 | 3 | 1 | 1 | Isomerases | 1 |
|  | 360 | 4 | 1 | 1 | Nucleic acids | 1 |
|  | 361 | 4 | 1 | 1 | Nuclear Receptors | 1 |
|  | 362 | 2 | 1 | 1 | GPCRs | 1 |
|  | 363 | 4 | 1 | 1 | Nuclear Receptors | 1 |
|  | 364 | 2 | 1 | 1 | Lyases | 1 |
|  | 365 | 6 | 1 | 1 | Nuclear Receptors | 1 |
|  | 366 | 2 | 1 | 1 | Ion Channels | 1 |
|  | 367 | 5 | 1 | 1 | Nuclear Receptors | 1 |
|  | 368 | 3 | 1 | 1 | Oxidoreductases | 1 |
|  | 369 | 5 | 1 | 1 | GPCRs | 1 |
|  | 370 | 2 | 1 | 1 | GPCRs | 1 |
|  | 371 | 4 | 1 | 1 | Nuclear Receptors | 1 |
|  | 372 | 3 | 1 | 1 | Isomerases | 1 |
|  | 373 | 3 | 1 | 1 | Others | 1 |
|  | 374 | 4 | 1 | 1 | Hydrolases | 1 |
|  | 375 | 2 | 1 | 1 | Oxidoreductases | 1 |
|  | 376 | 2 | 1 | 1 | Ion Channels | 1 |
|  | 377 | 1 | 1 | 1 | GPCRs | 1 |
|  | 378 | 1 | 1 | 1 | GPCRs | 1 |
|  | 379 | 2 | 1 | 1 | GPCRs | 1 |
|  | 380 | 2 | 1 | 1 | Nuclear Receptors | 1 |
|  | 381 | 1 | 1 | 1 | Ion Channels | 1 |
|  | 382 | 1 | 1 | 1 | Cellular proteins | 1 |
|  | 383 | 1 | 1 | 1 | Other Receptors | 1 |
|  | 384 | 6 | 1 | 1 | GPCRs | 1 |
|  | 385 | 3 | 1 | 1 | GPCRs | 1 |
|  | 386 | 3 | 1 | 1 | Cellular proteins | 1 |
|  | 387 | 2 | 1 | 1 | Nuclear Receptors | 1 |
|  | 388 | 3 | 1 | 1 | GPCRs | 1 |
|  | 389 | 1 | 1 | 1 | Other Receptors | 1 |
|  | 390 | 4 | 1 | 1 | Oxidoreductases | 1 |
|  | 391 | 2 | 1 | 1 | Ion Channels | 1 |
|  | 392 | 2 | 1 | 1 | Ion Channels | 1 |
|  | 393 | 2 | 1 | 1 | GPCRs | 1 |
|  | 394 | 3 | 1 | 1 | GPCRs | 1 |
|  | 395 | 2 | 1 | 1 | Lyases | 1 |
|  | 396 | 3 | 1 | 1 | Isomerases | 1 |
|  | 397 | 2 | 1 | 1 | Hydrolases | 1 |
|  | 398 | 4 | 1 | 1 | Nuclear Receptors | 1 |
|  | 399 | 2 | 1 | 1 | Nucleic acids | 1 |
|  | 400 | 2 | 1 | 1 | Isomerases | 1 |
|  | 401 | 3 | 1 | 1 | Ion Channels | 1 |
|  | 402 | 2 | 1 | 1 | Others | 1 |
|  | 403 | 4 | 1 | 1 | Other Receptors | 1 |
|  | 404 | 3 | 1 | 1 | GPCRs | 1 |
|  | 405 | 2 | 1 | 1 | GPCRs | 1 |
|  | 406 | 3 | 1 | 1 | GPCRs | 1 |
|  | 407 | 1 | 1 | 1 | Transferases | 1 |
|  | 408 | 1 | 1 | 1 | Others | 1 |
|  | 409 | 2 | 1 | 1 | Oxidoreductases | 1 |
|  | 410 | 4 | 1 | 1 | Nuclear Receptors | 1 |
|  | 411 | 1 | 1 | 1 | Others | 1 |
|  | 412 | 1 | 1 | 1 | Others | 1 |
|  | 413 | 2 | 1 | 1 | Transferases | 1 |
|  | 414 | 4 | 1 | 1 | Nuclear Receptors | 1 |
|  | 415 | 6 | 1 | 1 | Transferases | 1 |
|  | 416 | 4 | 1 | 1 | GPCRs | 1 |
|  | 417 | 3 | 1 | 1 | Ion Channels | 1 |
|  | 418 | 3 | 1 | 1 | GPCRs | 1 |
|  | 419 | 2 | 1 | 1 | Hydrolases | 1 |
|  | 420 | 4 | 1 | 1 | Nuclear Receptors | 1 |
|  | 421 | 3 | 1 | 1 | Ion Channels | 1 |
|  | 422 | 4 | 1 | 1 | GPCRs | 1 |
|  | 423 | 3 | 1 | 1 | Oxidoreductases | 1 |
|  | 424 | 3 | 1 | 1 | GPCRs | 1 |
|  | 425 | 1 | 1 | 1 | Penicillin binding proteins | 1 |
|  | 426 | 1 | 1 | 1 | Hydrolases | 1 |
|  | 427 | 3 | 1 | 1 | Other Receptors | 1 |
|  | 428 | 3 | 1 | 1 | GPCRs | 1 |
|  | 429 | 4 | 1 | 1 | GPCRs | 1 |
|  | 430 | 1 | 1 | 1 | Lyases | 1 |
|  | 431 | 6 | 1 | 1 | Nuclear Receptors | 1 |
|  | 432 | 2 | 1 | 1 | GPCRs | 1 |
|  | 433 | 5 | 1 | 1 | Others | 1 |
|  | | | | | | |
| ^a^Chemical structure of the fragment.  ^b^Unique numeric identifier for the fragment.  ^c^Number of contiguous rings in the fragment.  ^d^Frequency of the fragment; i.e. total number of times the structure appears in the drug set.  ^e^Number of drugs the fragment was found in; notice this number has to be equal or smaller than the frequency.  ^f^Protein target classes related to the drugs the fragment was obtained from.  ^g^Number of target classes related to the fragment. | | | | | | |
